# Density of host-seeking *Ixodes scapularis* nymphs by region, state, and county in the contiguous United States generated through national tick surveillance

**DOI:** 10.1016/j.ttbdis.2024.102316

**Published:** 2024-02-06

**Authors:** Erik Foster, Karen M. Holcomb, Rebecca J. Eisen

**Affiliations:** Division of Vector-Borne Diseases, National Center for Emerging and Zoonotic Infectious Diseases, Centers for Disease Control and Prevention, 3156 Rampart Road, Fort Collins, CO 80521, Fort Collins 80521, CO, USA

**Keywords:** Deer tick, Borrelia, Anaplasma, Babesia

## Abstract

The majority of vector-borne disease cases reported annually in the United States are caused by pathogens spread by the blacklegged tick, *Ixodes scapularis*. The number and geographic distribution of cases have increased as the geographic range and abundance of the tick have expanded in recent decades. A large proportion of Lyme disease and other *I. scapularis*-borne diseases are associated with nymphal tick bites; likelihood of such bites generally increases with increasing nymphal densities. National tick surveillance was initiated in 2018 to track changes in the distribution and abundance of medically important ticks at the county spatial scale throughout the United States. Tick surveillance records, including historical data collected prior to the initiation of the national program, are collated in the ArboNET Tick Module database. Through exploration of ArboNET Tick Module data, we found that efforts to quantify the density of host-seeking *I. scapularis* nymphs (DON) were unevenly distributed among geographic regions with the greatest proportion of counties sampled in the Northeast and Upper Midwest. Submissions covering tick collections from 2004 through 2022 revealed extensive variation in DON estimates at collection site, county, state, and regional spatial scales. Throughout the entire study period, county DON estimates ranged from 0.0 to 488.5 nymphs/1,000 m^2^. Although substantial variation was recorded within regions, DON estimates were greatest in the Northeast, Upper Midwest, and northern states within the Southeast regions (Virginia and North Carolina); densities were intermediate in the Ohio Valley and very low in the South and Northern Rockies and Plains regions. The proportion of counties classified as moderate or high DON was lower in the Northeast, Ohio Valley, and Southeast regions during the 2004 through 2017 time period (prior to initiation of the national tick surveillance program) compared to 2018 through 2022; DON estimates remained similarly low between these time periods in the South and the Northern Rockies and Plains regions. Despite the limitations described herein, the ArboNET Tick Module provides useful data for tracking changes in acarological risk across multiple geographic scales and long periods of time.

## 1. Introduction

Tick-borne illnesses are a persistent and increasing public health concern, accounting for the vast majority of reported vector-borne infections in the United States (U.S.). Lyme disease, caused primarily by *Borrelia burgdorferi* sensu stricto and spread by the blacklegged tick, *Ixodes scapularis*, is the most commonly reported tick-borne disease (Eisen et al., 2017; Rosenberg et al., 2018). *Ixodes scapularis* serves as a vector of several additional human pathogens including *Borrelia mayonii* (a less common Lyme disease agent in the Upper Midwest), *Anaplasma phagocytophilum* (anaplasmosis), *Babesia microti* (babesiosis), *Borrelia miyamotoi* (hard tick relapsing fever), *Ehrlichia muris eauclairensis* (ehrlichiosis), and Powassan virus disease (Powassan virus lineage II or deer tick virus that causes a neuroinvasive viral disease) (Eisen and Eisen, 2018). Over the past two decades, the geographic range of *I. scapularis* and associated human pathogens has expanded considerably, contributing to an increase in reported cases of tick-borne diseases, particularly in the Northeast, Ohio Valley, and Upper Midwest regions (Brinkerhoff et al., 2014; Kugeler et al., 2015; Lantos et al., 2017; Fleshman et al., 2022; Eisen and Eisen, 2023).

Effective prevention and diagnosis of tick-borne diseases hinge upon awareness by the public and healthcare professionals regarding when and where individuals are at risk for exposure to human-biting ticks and the pathogens they carry. Traditional public health surveillance that tracks the occurrence of notifiable tick-borne disease cases provides critical information to assess trends in where and when cases are reported but is currently limited in its ability to differentiate local exposures from travel-associated exposures to infected ticks. This is because human cases are reported to the patient home of residence, which may or may not be the site of exposure to infected ticks. Tick surveillance aims to supplement tick-borne disease surveillance by providing county estimates of the presence and abundance of medically important tick species, and presence and prevalence of associated human pathogens in human biting ticks (Eisen and Paddock, 2021). Previously, we updated maps showing the county level distributions of *I. scapularis* (Eisen et al., 2016; CDC, 2023), the pathogens they transmit (Fleshman et al., 2021, 2022) and the prevalence of pathogens in host-seeking nymphs and adults (Lehane et al., 2021; Foster et al., 2023).

Mapping the presence of medically important ticks and their associated human pathogens serves as a crucial initial step in defining the risk of acquiring tick-borne diseases. Additionally, gaining insight into regional variation in the density of host-seeking ticks provides useful information on the likelihood of human encounters with ticks across regions of the U.S. where ticks are established (Campbell et al., 1998; Falco and Fish, 1998; Pepin et al., 2012). Previous work demonstrated that *I. scapularis* nymphs are the most epidemiologically significant life stage in the transmission of *B. burgdorferi*. The timing of most human infections coincides with when nymphal ticks are host-seeking; nymphs are smaller than adults and more difficult to detect, potentially leading to a longer duration of feeding that is more likely to result in infection (Eisen, 2018; Mead, 2022). The host-seeking behavior of *I. scapularis* nymphs differs across the tick’s range in the eastern U.S., resulting in a more limited geographic range over which humans are likely to encounter questing nymphal ticks (Diuk-Wasser et al., 2010; Arsnoe et al., 2019; CDC, 2023). An earlier systematic effort to map the distribution and abundance of host-seeking *I. scapularis* nymphs was undertaken nearly two decades ago (Diuk-Wasser et al., 2006, 2010). Recent dramatic changes in the tick’s reported geographic range necessitated an update (Eisen et al., 2016; Eisen and Eisen, 2023).

Here, we assessed geographic and temporal trends in the collection of *I. scapularis* nymphal density data submitted to the U.S. Centers for Disease Control and Prevention (CDC) ArboNET Tick Module. The module was created in 2018 to house data collected as part of national tick surveillance efforts that were initiated that year (Eisen and Paddock, 2021; CDC, 2018), but historical records were also included in the database. Based on ArboNET Tick Module submissions, we report county, state, and regional variation in the density of host-seeking *I. scapularis* nymphs in the eastern U.S. and compare regional density estimates during two surveillance time periods: 2004 through 2017 (prior to initiation of the national tick surveillance program) and 2018 through 2022 (records collected as part of national tick surveillance efforts).

## 2. Methods

### 2.1. Density datasets

County, state, and regional estimates of *I. scapularis* nymphs per 1000 m^2^ (density of nymphs, DON) were derived from ArboNET Tick Module records of questing ticks collected from vegetation. ArboNET is the U.S. national arthropod-borne diseases surveillance system. In addition to reports of human disease cases, ArboNET maintains ecologic surveillance data on infections in vectors and host animals. Tick and tick-borne pathogen records are reported to the ArboNET Tick Module by U.S. state public health agencies representing the surveillance efforts of their programs and the programs of their academic and organizational partners. The Tick Module was added to ArboNET in 2018 to accept standardized records of tick presence and abundance, and tick-borne pathogen presence and prevalence. Records include the site and date of tick collections, the number of ticks collected by species and life stage, collection methods used (e.g., distances if conducting density sampling), and tick-borne pathogen testing results, if applicable (CDC, 2018).

**Fig. 1.**
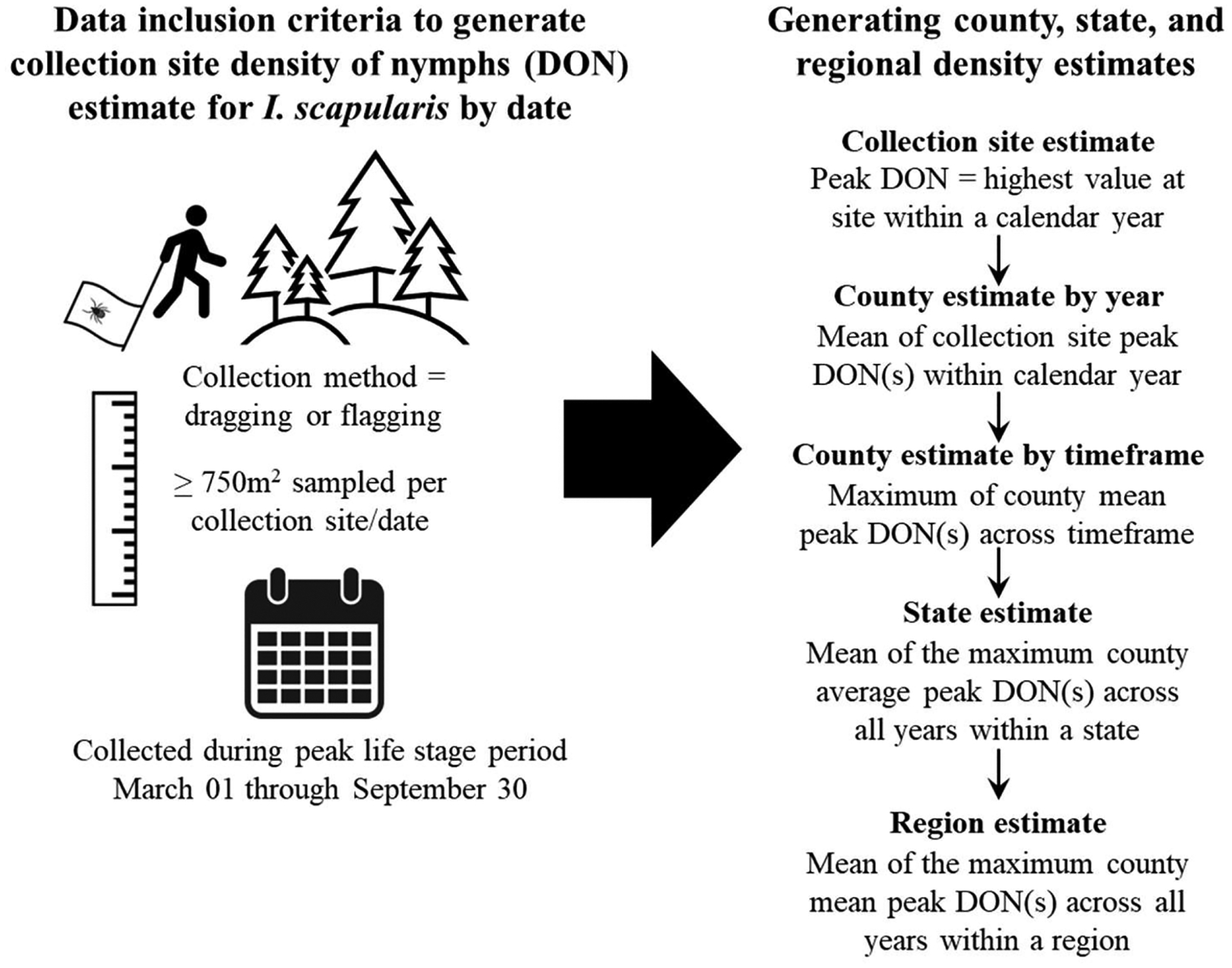
Study data inclusion and density estimate criteria. Collection site density estimates for nymphal *I. scapularis* were generated for sites where collection methods were flagging or dragging, distance flagged/dragged within a single date was ≥750m^2^, and collections were made during the presumed nymphal host-seeking period of March 01 through September 30 annually. Collection site peak density is defined as the highest value calculated for the site within a calendar year. To generate county-level estimates by year, the annual collection site peak estimates within a county were averaged for each year. County estimates for the entire study period (2004–2022), pre-ArboNET period (2004–2017), and ArboNET period (2018–2022) were generated by taking the maximum of the county average peak densities across each timeframe. State and regional estimates were generated only for the entire study period and are the average of the maximum county average peak densities across all years within the state or region.

Although the national tick surveillance program began in 2018, several CDC-funded tick surveillance efforts were conducted prior to the development of ArboNET Tick Module (e.g., Diuk-Wasser et al., 2010, 2012; Johnson et al., 2017, 2018). Data from those studies and others conducted from 2004 through 2017 were uploaded to ArboNET and represent historical records. In this analysis, we included records from any collection site and date where at least 750 m^2^ was sampled by dragging or flagging (CDC, 2018) during the *I. scapularis* nymphal host-seeking season, defined here as March 1 through September 30. To derive the collection site-specific peak DON per year if multiple sampling visits met the data inclusion criteria, we selected the sampling observation with the maximum DON (Fig. 1). In instances where a site was sampled only once in a given year, the single estimate was recorded as the site-specific peak DON. Collection site data were aggregated by year for counties, states, and regions as described below (Fig. 1).

For each calendar year in which sampling was conducted, we calculated the mean of site-specific peak DONs within each county to derive an annual estimate of the county’s average peak DON. In the majority of counties and years, only a single site was sampled per county, resulting in the same estimate for maximum and average annual peak DON. In instances where multiple sites were sampled per year, we aimed to smooth outliers by taking the county average peak DON per year, rather than selecting only the maximum value. To avoid underestimating the recorded average peak DON, particularly in counties that were sampled longitudinally through a period of emergence of *I. scapularis*, we report the county specific maximum annual average peak DON across all years sampled (2004 through 2022), for the pre-ArboNET Tick Module time period (2004 through 2017), and for the ArboNET Tick Module period (2018 through 2022). The state specific average was derived from the mean of county specific maximum peak DONs across each county in the state. The regional average was derived using the National Oceanic and Atmospheric Administration (NOAA) climate regions (Karl and Koss, 1984) by averaging each county specific maximum peak DON across each county within a region (Fig. 1). Range values reflect the minimum and maximum of peak DON values observed at the collection site level within the region of interest across all observation years. We assigned a geographic representativeness (GR) score to states and regions, which is the proportion of counties within the state or region where *I. scapularis* nymphal density was estimated.

### 2.2. Comparison of DON by time period and region

We classified non-zero county-level DON estimates by quartile for the entire study period (2004 through 2022) and applied the classification scheme to the 2004 through 2017, and 2018 through 2022 time periods. This resulted in four categories: density estimates of zero or within the lower-, inter- or upper-quartile ranges. To evaluate regional changes in nymphal density between time periods, we combined the zero and lower quartile counties (“low”) and the interquartile and upper quartile counties (“moderate or high”). We performed contingency table analyses in JMP (JMP, Version 16, SAS Institute Inc., Cary, NC) to compare the distribution of counties classified in these two categories between timeframes per region.

We then broadly compared nymphal densities between regions. Using the full time period (2004–2022), we compared the distribution of counties classified as “low” and “moderate or high” across regions. For this, we performed *χ*^2^ tests with a Bonferroni correction to adjust for multiple comparisons (i.e., all pairwise comparisons between regions).

### 2.3. Mapping

Tables containing nymphal *I. scapularis* county density values for the 2004 through 2017 and 2018 through 2022 time periods, and the entire study period (2004 through 2022), were joined to a U.S. census county-level geographic information system (GIS) layer in ArcMap (version 8.2, ESRI, Redlands, CA) using Federal Information Processing Standards (FIPS) codes. For each county with an estimate, we plotted DON according to interquartile categories (i.e., zero, lower 25 %, middle 50 %, and upper 25 %) for each study period. Additionally, tables containing county presence classifications for *I. scapularis* (CDC, 2023) were added to the GIS layer to contrast the range of reported *I. scapularis* presence to counties where density estimates have been reported in ArboNET. County, state, and NOAA climate region outlines were added for visualization.

## 3. Results

### 3.1. Geographic representativeness of DON estimates for the entire study period (2004 through 2022)

**Fig. 2.**
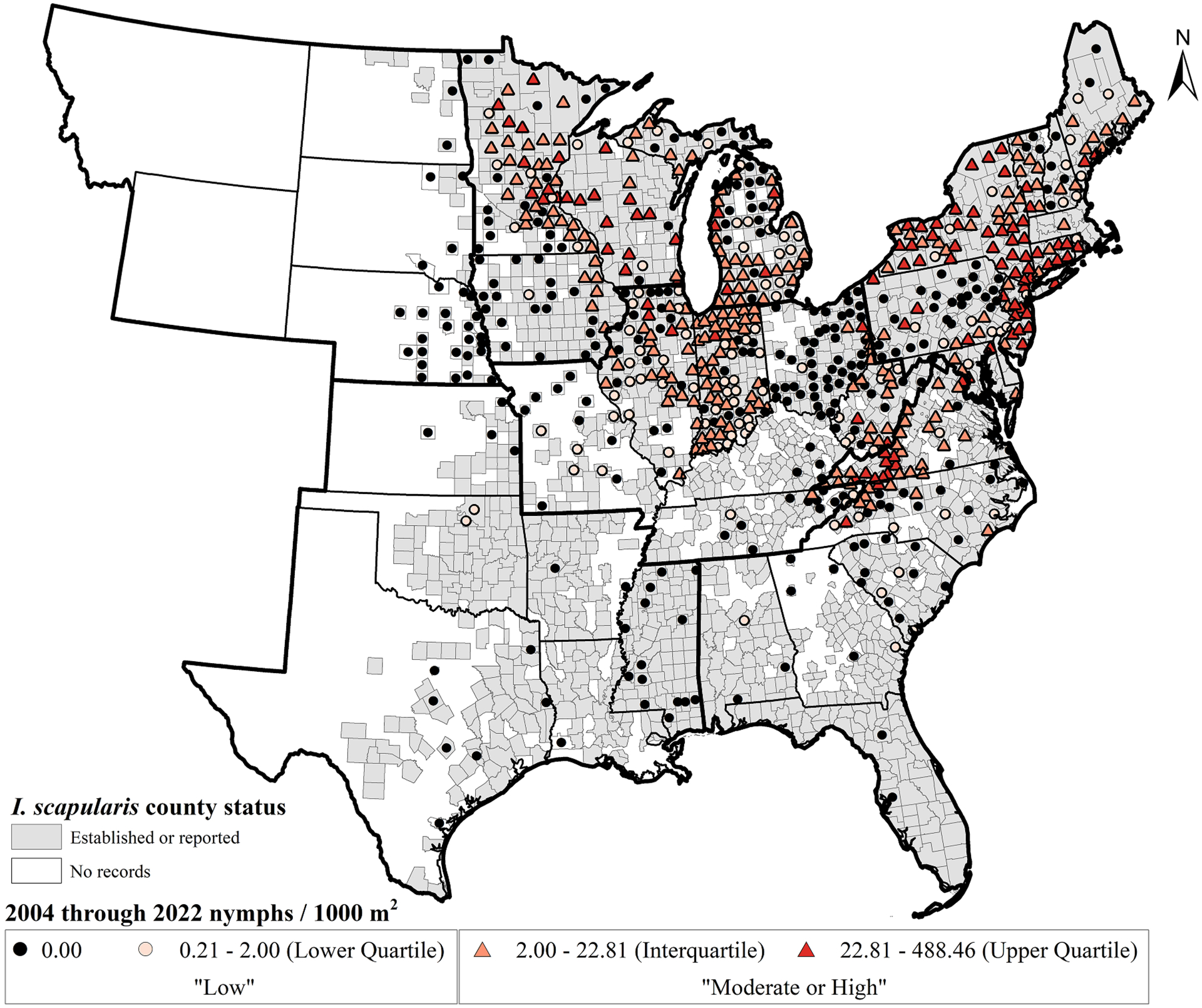
Reported county estimates of mean peak density of host-seeking nymphal *I. scapularis* (nymphs/1000m^2^) from (A) 2004 through 2017 (pre-ArboNET), and (B) 2018 through 2022 (ArboNET) in regions of the contiguous U.S. where *I. scapularis* has been documented (CDC, 2023). County density estimates indicated by colored circles (for densities in the “low” category) or triangles (for densities classified as “moderate or high”). Counties with documented presence of *I. scapularis* are shaded dark gray (CDC, 2023). Inset - National Oceanic and Atmospheric Administration (NOAA) climate regions represented in this study for the contiguous United States. Regions denoted by bold outline and region abbreviation: Northeast (NE), Northern Rockies and Plains (NRP), Ohio Valley (OV), South (South), Southeast (SE), Upper Midwest (UMW). Adapted from Karl and Koss (1984).

**Fig. 3.**
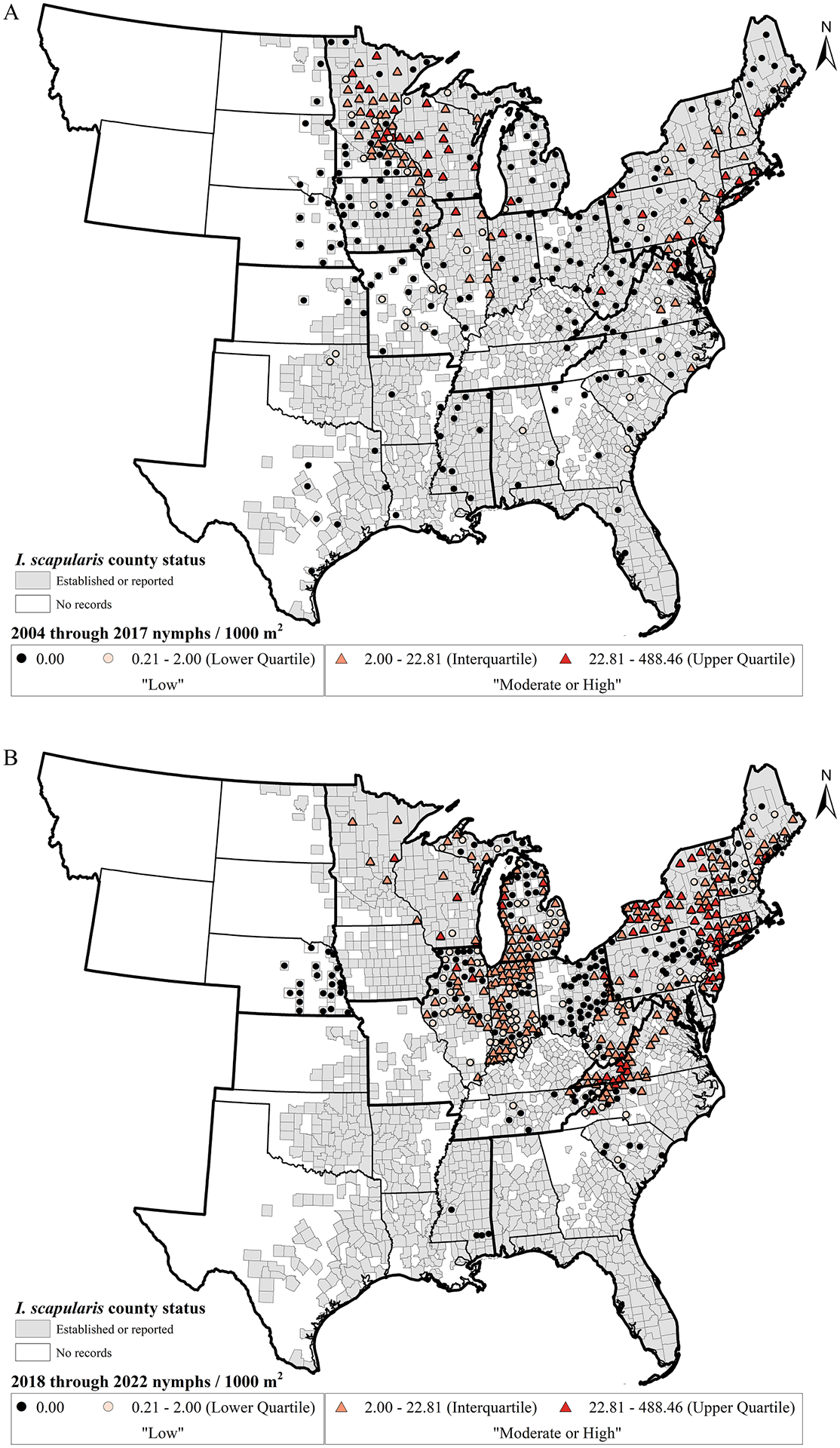
Reported county estimates of mean peak density of host-seeking nymphal *I. scapularis* (nymphs/1000m^2^) from (A) 2004 through 2017 (pre-ArboNET), and (B) 2018 through 2022 (ArboNET) in regions of the contiguous U.S. where *I. scapularis* has been documented (CDC, 2023). County density estimates indicated by colored circles (for densities in the “low” category) or triangles (for densities classified as “moderate or high”). Quartiles of non-zero density estimates were classified based on the full 2004 through 2022 dataset for comparison across timeframes. Counties with documented presence of *I. scapularis* are shaded dark gray (CDC, 2023). Inset - National Oceanic and Atmospheric Administration (NOAA) climate regions represented in this study for the contiguous United States. Regions denoted by bold outline and region abbreviation: Northeast (NE), Northern Rockies and Plains (NRP), Ohio Valley (OV), South (South), Southeast (SE), Upper Midwest (UMW). Adapted from Karl and Koss (1984).

Tick surveys focused on the eastern half of the U.S. where *I. scapularis* is widely distributed (Figs. 2 and 3). From 2004 through 2022, the proportion of counties surveyed for *I. scapularis* ranged from 16 % (101 counties) in the South region to 78 % (193 counties) in the Northeast region with a total of 1221 U.S. counties surveyed, representing 45.4 % of counties in the eastern U.S. (Table 1). A total of 2542 collection sites met study inclusion criteria for generating DON estimates, resulting in 720 counties with DON estimates for the entire study period (Table 1, Fig. 2). Of these 720 counties, 488 (67.8 %) included only a single collection site per year. The median number of sites where density sampling was conducted per county per year was 1 (range 1–19 sites). A majority of counties (*n* = 402; 55.8 %) included only a single year of nymphal density sampling; density sampling was conducted for a median of one (range 1–14 years) year per county. Thus, the majority of county DON estimates from 2004 to 2022 were based on sampling a single site within the county during only one of eighteen years. Within each county, the median number of sampling visits per collection year was 3 (range 1–37 visits).

The GR of counties with density estimates varied widely among states and regions within the geographic range of *I. scapularis* (Table 1; Figs. 2–3). Overall, the GR of nymphal density estimates was highest in the Northeast (64.5 % of counties in the Northeast, range 0.0–100.0 % of counties per state in the Northeast), Upper Midwest (51.9 % of counties in the Upper Midwest, range 26.3–88.0 % of counties per state in the Upper Midwest), and Ohio Valley (36.0 % of counties in the Ohio Valley, range 4.2–78.3 % of counties per state in the Ohio Valley) regions. In contrast, the Southeast (15.0 % of counties in the Southeast, range 3.0–28.3 % of counties per state in the Southeast), Northern Rockies and Plains (10.0 % of counties in the Northern Rockies and Plains, range 0.0–24.7 % of counties in the Northern Rockies and Plains), and South (4.6 % of counties in the South, range 1.6–17.1 % of counties per state in the South) regions had the lowest GR of the regions in the study that reported density estimates. Of note, a majority of non-zero county DON estimates in the Southeast region were derived from northernmost states in that region, including Virginia and North Carolina (Table 1, Fig. 2).

### 3.2. Geographic representativeness of DON estimates prior to initiation of national tick surveillance (2004 through 2017)

From 2004 to 2017, prior to the initiation of national tick surveillance efforts, 565 sites across 328 counties were sampled adequately to estimate DON. ArboNET data from this period are dominated by three CDC-funded surveillance efforts in the Upper Midwestern state of Minnesota (Johnson et al., 2018), U.S. National Parks in the Northeast (Johnson et al., 2017) and across the entire eastern U.S. (Diuk-Wasser et al., 2010). Among the 328 counties for which DON was estimated, 295 (89.9 %) county estimates were based on a single collection site per year. Density sampling was conducted at a median of one (range 1–6 sites) site per county per year. A total of 236 of 328 counties (72.0 %) included only a single year of nymphal density sampling. Density sampling was conducted within a county for a median of one (range 1–11 years) year. Within each county, a median of five (range 1–8 visits) sampling visits per collection year were performed.

Overall, the GR of DON estimates were highest in the Upper Midwest (35.2 % of counties in the Upper Midwest, range 22.2–66.7 % of counties per state in the Upper Midwest), Northeast (22.5 % of counties in the Northeast, range 0.0–40.0 % of counties per state in the Northeast), and Ohio Valley (10.6 % of counties in the Ohio Valley, range 1.1–20.0 % of counties per state in the Ohio Valley) regions. In contrast, the Southeast (7.3 % of counties in the Southeast, range 3.1–15.2 % of counties per state in the Southeast), Northern Rockies and Plains (4.8 % of counties in the Northern Rockies and Plains, range 0.0–8.6 % of counties per state in the Northern Rockies and Plains), and South (4.0 % of counties in the South, range 1.6–12.2 % of counties per state in the South) regions had the lowest GR of the regions in the study that reported density estimates (Table 2, Fig. 3).

### 3.3. Geographic representativeness of DON estimates during national tick surveillance period (2018 through 2022)

From the initiation of national tick surveillance onward (2018 through 2022), 1938 sites were adequately sampled to estimate DON resulting in 500 counties with estimates. This represents a more than tripling of the number of sites and about a third more counties sampled in the most recent five years compared with the 19 years prior. From 2018 through 2022, 298 (59.6 %) of 500 counties had county DON estimates based on only one collection site per year. Density sampling was conducted at a median of one (range 1–19 sites) site per county per year. A total of 290 (58.0 %) of 500 county estimates across the 2018 through 2022 time period included only a single year of nymphal density sampling and the median number of years where density sampling was conducted within a county was 1 (range 1–5 years). Within each county, the median number of sampling visits per collection year was 2 (range 1–49 sampling visits per year).

Overall, the GR of nymphal density estimates was highest in the Northeast (56.7 % of counties in the Northeast, range 0.0–100.0 % of counties per state in the Northeast), Ohio Valley (30.0 % of counties in the Ohio Valley, range 0.0–77.2 % of counties per state in the Ohio Valley), and Upper Midwest (24.3 % of counties in the Upper Midwest, range 0.0–85.5 % of counties per state in the Upper Midwest) regions. In contrast, the Southeast (9.1 % of counties in the Southeast, range 0.0–22.6 % of counties per state in the Southeast), Northern Rockies and Plains (7.6 % of counties in the Northern Rockies and Plains, range 0.0–23.7 % of counties per state in the Northern Rockies and Plains), and South (0.6 % of counties in the South, range 0.0–4.9 % of counties per state in the South) regions had the lowest GR of the regions in the study that reported density estimates (Table 2, Fig. 3).

### 3.4. Geographic variation in DON, 2004 through 2022

Estimates of DON varied widely at all geographic scales from collection site to region. Throughout the entire study period, county DON estimates ranged from 0.0 nymphs/1000 m^2^ to 488.5 nymphs/1000 m2 (Fig. 2). Average state nymphal density values ranged from 0.0 nymphs/1000 m2 in many southern and northern Rockies and Plains states, to 66.9 nymphs/1000 m2 (range 0.0–313.0 nymphs/1000 m2) in Wisconsin. Regional nymphal density values ranged from 0.0 nymphs/1000 m2 in the Northern Rockies and Plains region to 24.7 nymphs/1000 m2 (range 0.0–488.5 nymphs/1000 m2) in the Southeast region (Table 1).

Considering the full 18-year period, the Northern Rockies and Plains and South regions each had all counties classified within the low category (Table 2). The proportions of counties in the Southeast, Upper Midwest, and Northeast regions categorized as moderate or high was similar: 47.7 % (41 of 86 Southeastern counties), 52.0 % (92 of 177 Upper Midwestern counties), and 63.9 % (101 of 158 Northeastern counties) (*χ*^2^≤6.044, adjusted *p*-value≥0.21). The Ohio Valley region contained a lower percentage of counties classified as moderate or high (35.0 %; 84 of 240), but this was not significantly different than the Southeast region (χ^*2*^_(1, 326)_ =4.30, adjusted *p* = 0.57).

### 3.5. Regional comparison of DON between time periods: 2004 through 2017 vs 2018 through 2022

The proportion of counties classified as moderate or high was significantly greater during the 2018 through 2022 time period compared with 2004 through 2017 in the Northeast (χ^*2*^_(1, 94)_ =5.01, *p* = 0.025), Ohio Valley (χ^*2*^_(1271)_ =11.54, *p<*0.001), and Southeast (χ^*2*^_(1,94)_ =32.25, *p<*0.0001) regions. The Northern Rockies and Plains, South, and Upper Midwest regions showed no significant differences in the proportions of counties assigned to the moderate or high DON categories between time periods (Table 2, Fig. 3).

## 4. Discussion

Our review of ArboNET Tick Module submissions covering tick collections from 2004 through 2022 revealed extensive variation in estimates of the density of host-seeking *I. scapularis* nymphs at collection site, county, state, and regional spatial scales. The high degree of variation reported at larger spatial scales was expected and is consistent with previous studies that reported substantial differences in nymphal densities among sampling sites even across very small distances, within a single day of sampling, or across seasons (Pardanani and Mather, 2004; Jordan et al., 2022; Schulze and Jordan, 2022). Variation in DON is attributable to the tick’s response to weather conditions, host availability, seasonality of host-seeking, site characteristics, and sampling effort (Schulze et al., 1997; Schulze and Jordan, 2003; Allan et al., 2003; Rand et al., 2003; Ostfeld et al., 2006; Dobson, 2013, 2014; Kilpatrick et al., 2014; Burtis et al., 2016, 2019, 2023; Borgmann-Winter and Allen, 2020; Ginsberg et al., 2020). Although variation in reported DON was considerable among counties, we show that average DON estimates are greatest in the Northeast, Upper Midwest, and northernmost states in the Southeast (Virginia and North Carolina) regions; densities were intermediate in the Ohio Valley and very low in the South and Northern Rockies and Plains regions. Despite the overall higher densities observed in northern states of the eastern U.S., DON estimates ranged from zero nymphs to as many as 488.5 nymphs per 1000 m2 among collection sites. The public health community and the public should be aware that the likelihood of encountering *I. scapularis* nymphs is spatially and temporally dynamic, but risk of encounters can be significant in these areas.

Though *I. scapularis* has been recorded as present in numerous counties in the South and southern states within the Southeast regions (Eisen et al., 2016; Figs. 2 and 3), in counties where drag sampling was conducted and data were submitted to ArboNET, no nymphs or very few were collected. As a result, risk of human encounters with *I. scapularis* nymphs in these areas is expected to be very low. In previously published maps, several counties lacked records of the tick’s presence in the Northern Rockies and Plains region (Eisen et al., 2016; CDC, 2023). However, maps of tick presence do not typically account for sampling effort, making it difficult to determine if the tick is truly absent or if surveillance was not conducted. Recent drag sampling efforts reported here suggest that encountering nymphal ticks is rare, even in the eastern counties of the Northern Rockies and Plains region where the tick has been reported previously. Despite sampling at least 750 m2 at 94 sites from 29 counties in Nebraska, North Dakota and South Dakota, no nymphs were collected from 2004 through 2022. However, it is important to highlight that data inclusion criteria for this study required drag or flag sampling of at least 750 m2 per collection site within fixed time periods and therefore, observations were excluded (Table 1). In some instances, host-seeking nymphs were collected by drag sampling within smaller sampling grids or using timed collections. Such observations are depicted in the tick presence maps presented here, but densities were not estimated. Nonetheless, these efforts demonstrate that questing nymphs are present and sometimes abundant within limited forested locations in the eastern reaches of the Northern Rockies and Plain region (Russart et al., 2014; Nielsen et al., 2020; Black et al., 2021). For these reasons, it was not possible within the scope of the limited dataset presented here to compare absence and presence of *I. scapularis* over time.

A recent review described changes in the tick’s distribution with significant recolonization of counties in the Upper Midwest, Ohio Valley, Northeast, and northern states in the Southeast region over the past half century (Eisen and Eisen, 2023). Numerous small-scale studies have noted trends of increasing DON immediately after colonization, then stabilization in long-term density estimates (Hamer et al., 2010; Serra et al., 2013; Brinkerhoff et al., 2014; Tran et al., 2021; Christie et al., 2022). As a result of these tick colonization and establishment processes, it was not surprising to see differences in the proportions of counties classified as moderate or high between time periods. The proportion of counties classified as moderate or high DON was greater in the Northeast, Ohio Valley, and Southeast regions from 2018 through 2022 compared with 2004 through 2017; DON estimates remained similarly low between these time periods in the South and the Northern Rockies and Plains regions. The timeframes examined in this study are somewhat arbitrary relative to range expansion of the tick. Instead, they were derived based on the timing of establishment of the national tick surveillance program and the ArboNET Tick Module database. In addition, the sampling strategy was not optimized to detect changes in DON at small spatial scales. For the most part, the same sites were not sampled repeatedly, and county estimates were often derived from different collection sites over separate years. These factors, along with describing trends at regional scales, may have obscured real changes in nymphal densities over time that were reported in previously published studies. For example, the number of counties classified in the moderate or high DON category was similar between time periods in the Upper Midwest. However, previous studies showed significant expansion of the tick’s range and local increases in DON in newly colonized areas mostly prior to 2018 in Iowa (Oliver et al., 2017), Minnesota (Diuk-Wasser et al., 2010; Johnson et al., 2018), Wisconsin (Lee et al., 2013), Indiana (Pinger et al., 1996), Michigan (Hamer et al., 2010; Lantos et al., 2017), and Ohio (Wang et al., 2014). Owing to the lack of consistent sampling effort over time and space, we are limited in our ability to use ArboNET Tick Module data to track changes in DON at fine spatial and temporal scales. However, we were able to detect significant differences in DON categories at coarse (regional) spatial scales and between broad temporal periods, but differences in sampling effort (represented in part by the GR scores) should be taken into consideration when viewing these records.

Although the density of host-seeking *I. scapularis* is generally considered a critical component in estimating acarological risk for exposure to Lyme disease spirochetes and other *I. scapularis*-borne pathogens (Mather et al., 1996; Falco and Fish, 1998; Campbell et al., 1998; Diuk-Wasser et al., 2010, 2012; Pepin et al., 2012), it is a notoriously difficult value to measure precisely. Previous studies have highlighted significant variability in estimates at the community level (Dobson 2013; Pardanani and Mather, 2004; Hahn et al., 2018; Schulze and Jordan, 2022). The precision of DON estimates are greatly improved by repeated drag sampling of the same sites on multiple occasion throughout the year (Diuk-Wasser et al., 2006; Dobson, 2013). Understanding spatial trends in DON is improved by careful selection of sampling sites (Diuk-Wasser et al., 2006, 2010; Johnson et al., 2018). Although such stringent criteria were met in several research studies included in historical ArboNET data (e.g.; Diuk-Wasser et al., 2010; Johnson et al., 2018), adherence to such costly and labor-intensive implementation schemes are rarely achieved in public health surveillance (Mader et al., 2021). We found that efforts to quantify DON were unevenly distributed among geographic regions with the greatest proportion of counties sampled being in the Northeast and Upper Midwest. The majority of county DON estimates from 2004 through 2022 were based on sampling a single site within the county only a single year across the observation period. The median number of visits per site per year to estimate DON declined from 5 visits per year from 2004 through 2017 to only 2 visits per year from 2018 through 2022. However, despite declines in the intensity of sampling per collection location, the proportion of counties sampled within regions vastly increased from after 2018 in the Northeast, Southeast, Northern Rockies and Plains, and Ohio Valley. We did not observe large changes in effort between time periods in the remaining regions.

Given the limitations of sampling conducted as part of national tick surveillance efforts, we cannot estimate DON with precision at fine spatial and temporal scales. Comparisons between time periods can be difficult based on inconsistent sampling and high variation in DON estimates. Despite these limitations, ArboNET Tick Module data provide useful information for tracking changes in acarological risk categories across broad geographic scales and long periods of time. Such efforts aid in monitoring expansion in the distribution of host-seeking nymphs, particularly into the Southeast where human encounters with *I. scapularis* nymphs and Lyme disease cases have each steadily increased over the past two decades (Brinkerhoff et al., 2014; Lantos et al., 2015, 2021).

## Figures and Tables

**Table 1:** Regional and state density estimates for host-seeking *I. scapularis* nymphs (no. nymphs/1000 m^2^)^a^.

Region^b^ / State^c^	Density estimate using max of average peak county density^d^	Minimum and maximum observed site-level density	No. counties sampled meeting inclusion criteria (%)	No. surveillance sites meeting inclusion criteria	No. counties sampled but not-meeting inclusion criteria (%)
*Northeast*	24.25	0.00 – 314.47	158 (64 %)	1007	35 (14 %)
Connecticut	30.95	0.00 – 137.33	8 (100 %)	165	0 (0 %)
Deleware	*No records*	N/A	0 (0 %)	0	0 (0 %)
District of Columbia	111.11	0.00 – 111.11	1 (100 %)	23	0 (0 %)
Maine	11.92	0.00 – 114.16	16 (100 %)	116	0 (0 %)
Maryland	15.21	0.00 – 56.00	6 (25 %)	48	2 (8 %)
Massachusetts	22.00	5.00 – 22.00	1 (7 %)	4	0 (0 %)
New Hampshire	1.17	0.00 – 12.48	10 (100 %)	76	0 (0 %)
New Jersey	33.05	1.00 – 124.00	14 (67 %)	41	6 (29 %)
New York	46.95	0.00 – 314.47	48 (77 %)	303	0 (0 %)
Pennsylvania	5.83	0.00 – 89.17	40 (60 %)	83	27 (40 %)
Rhode Island	23.00	1.00 – 45.00	2 (40 %)	3	0 (0 %)
Vermont	13.52	0.00 – 257.33	12 (86 %)	145	0 (0 %)
*Northern Rockies and Plains*	0.00	0.00 – 0.00	29 (10 %)	94	10 (3 %)
Montana	*No records*	N/A	0 (0 %)	0	0 (0 %)
Nebraska	0.00	0.00 – 0.00	23 (25 %)	43	3 (3 %)
North Dakota	0.00	0.00 – 0.00	2 (4 %)	5	2 (4 %)
South Dakota	0.00	0.00 – 0.00	4 (6 %)	7	5 (8 %)
Wyoming	*No records*	N/A	0 (0 %)	0	1 (4 %)
*Ohio Valley*	3.76	0.00 – 53.33	240 (36 %)	583	137 (21 %)
Illinois	4.89	0.00 – 46.67	57 (56 %)	110	2 (2 %)
Indiana	4.80	0.00 – 35.00	72 (78 %)	242	1 (1 %)
Kentucky	0.00	0.00 – 0.00	5 (4 %)	6	27 (23 %)
Missouri	0.36	0.00 – 1.00	14 (12 %)	15	35 (30 %)
Ohio	0.91	0.00 – 53.33	46 (52 %)	57	33 (38 %)
Tennessee	1.00	0.00 – 11.63	14 (15 %)	43	19 (20 %)
West Virginia	6.79	0.00 – 40.00	32 (58 %)	110	20 (36 %)
*South*	0.10	0.00 – 2.00	30 (5 %)	39	71 (11 %)
Arkansas	0.00	0.00 – 0.00	2 (3 %)	2	1 (1 %)
Kansas	0.00	0.00 – 0.00	4 (4 %)	4	13 (12 %)
Louisiana	0.00	0.00 – 0.00	1 (2 %)	1	8 (13 %)
Mississippi	0.00	0.00 – 0.00	14 (17 %)	23	34 (41 %)
Oklahoma	1.50	1.00 – 2.00	2 (3 %)	2	14 (18 %)
Texas	0.00	0.00 – 0.00	7 (3 %)	7	1 (0 %)
*Southeast*	24.67	0.00 – 488.46	86 (15 %)	153	110 (19 %)
Alabama	0.33	0.00 – 1.00	3 (4 %)	3	24 (36 %)
Florida	0.00	0.00 – 0.00	2 (3 %)	2	26 (39 %)
Georgia	0.20	0.00 – 1.00	5 (3 %)	5	35 (22 %)
North Carolina	7.05	0.00 – 120.00	26 (26 %)	55	10 (10 %)
South Carolina	0.25	0.00 – 1.31	13 (28 %)	14	10 (22 %)
Virginia	52.23	0.00 – 488.46	37 (28 %)	74	5 (4 %)
*Upper Midwest*	15.56	0.00 – 313.00	177 (52 %)	666	54 (16 %)
Iowa	2.77	0.00 – 20.00	26 (26 %)	33	41 (41 %)
Michigan	6.86	0.00 – 91.25	73 (88 %)	375	0 (0 %)
Minnesota	14.54	0.00 – 272.00	58 (67 %)	204	11 (13 %)
Wisconsin	66.88	0.00 – 313.00	20 (28 %)	54	2 (3 %)

a Host seeking *Ixodes scapularis* collected by public health agencies and academic partners. Cumulative density estimates generated from data submitted to the U.S. Centers for Disease Control and Prevention (CDC) ArboNET Tick Module, and from studies where CDC was the tickborne pathogen testing agency (2004–2022). Density of *I. scapularis* nymphs was calculated where any site was sampled >750m^2^ during a single visit and during peak nymphal season according to CDC Guidelines: “Surveillance for Ixodes scapularis and pathogens found in this tick species in the United States” (2018). https://www.cdc.gov/ticks/resources/TickSurveillance_Iscapularis-P.pdf.

b Regions correspond to U.S. Climate Regions defined by the U.S. National Oceanic and Atmospheric Administration (NOAA), National Centers for Environmental Information.

c Values reported only for states where at least one collection site met data inclusion criteria to estimate nymphal density for the study period.

d Density estimates using the maximum of the average peak state specific average were derived from the mean of county specific maximum peak DONs across each county in the state.

**Table 2:** Distribution of county quartile nymphal *I. scapularis* density classification by region^a^. Distribution of counties falling within zero to lower quartile (“low”) and inter- to upper quartiles (“moderate to high”) were compared between regions for the whole time period (2004–2022) and between time periods per region using χ^*2*^ tests.

		*Contingency analysis “low”*		*Contingency analysis “moderate or high”*	
Region^b^	No. counties (%) with density estimate^c^	No. counties (%) zero	No. counties (%) lower quartile	No. counties (%) interquartile	No. counties (%) upper quartile
Northeast (2004–2022)^A^	158 (64.49 %)	38 (24.05 %)	19 (12.03 %)	42 (26.58 %)	59 (37.34 %)
* 2004–2017	55 (22.45 %)	25 (45.45 %)	4 (7.27 %)	14 (25.45 %)	12 (21.82 %)
2018–2022	139 (56.73 %)	32 (23.02 %)	17 (12.23 %)	40 (28.78 %)	50 (35.97 %)
Northern Rockies and Plains (2004–2022)^B^	29 (9.97 %)	29 (100.00 %)	0 (0.00 %)	0 (0.00 %)	0 (0.00 %)
^NS^ 2004–2017	14 (4.81 %)	14 (100.00 %)	0 (0.00 %)	0 (0.00 %)	0 (0.00 %)
2018–2022	22 (7.56 %)	22 (100.00 %)	0 (0.00 %)	0 (0.00 %)	0 (0.00 %)
Ohio Valley (2004–2022)^C^	240 (35.98 %)	104 (43.33 %)	52 (21.67 %)	77 (32.08 %)	7 (2.92 %)
** 2004–2017	71 (10.64 %)	51 (71.83 %)	8 (11.27 %)	9 (12.68 %)	3 (4.23 %)
2018–2022	200 (29.99 %)	75 (37.50 %)	47 (23.50 %)	74 (37.00 %)	4 (2.00 %)
South (2004–2022)^B^	30 (4.57 %)	28 (93.33 %)	2 (6.67 %)	0 (0.00 %)	0 (0.00 %)
^NS^ 2004–2017	26 (3.96 %)	24 (92.31 %)	2 (7.69 %)	0 (0.00 %)	0 (0.00 %)
2018–2022	4 (0.61 %)	4 (100.00 %)	0 (0.00 %)	0 (0.00 %)	0 (0.00 %)
Southeast (2004–2022)^AC^	86 (15.03 %)	34 (39.53 %)	11 (12.79 %)	29 (33.72 %)	12 (13.95 %)
*** 2004–2017	42 (7.34 %)	29 (69.05 %)	7 (16.67 %)	5 (11.90 %)	1 (2.38 %)
2018–2022	52 (9.09 %)	10 (19.23 %)	4 (7.69 %)	27 (51.92 %)	11 (21.15 %)
Upper Midwest (2004–2022)^A^	177 (51.91 %)	60 (33.90 %)	25 (14.12 %)	64 (36.16 %)	28 (15.82 %)
^NS^ 2004–2017	120 (35.19 %)	55 (45.83 %)	10 (8.33 %)	34 (28.33 %)	21 (17.50 %)
2018–2022	83 (24.34 %)	20 (24.10 %)	17 (20.48 %)	38 (45.78 %)	8 (9.64 %)

a Regions correspond to U.S. Climate Regions defined by the U.S. National Oceanic and Atmospheric Administration (NOAA), National Centers for Environmental Information.

b Regions with the same letter are not different in the distribution of counties categorized as “low” and “moderate or high” for 2004–2022 by a *χ*^2^ test with a Bonferroni correction for multiple comparisons. Non-zero county-level DON estimates were classified by quartile for the entire study period (2004 through 2022) and applied to the 2004 through 2017, and 2018 through 2022 time periods.

c Total number of counties with density estimates for the entire time period will not equal the sum of counties from pre-ArboNET (2004–2017) and ArboNET (2018–2022) time period, as there are some counties with estimates generated in both time frames (*N* = 108 total). Percentages relative to number of counties per region.

* *P*-value from *χ*^2^ test of distribution of counties categorized as “low” and “moderate to high” within a region for 2004–2017 and 2018–2022, NS: not significant, *P<0.05, **P<0.001, ***P < 0.0001. Note that all significant tests had an increase in the proportion of counties in the “moderate to high” classification between time periods.

## Data Availability

Data sharing agreements in ArboNET preclude sharing data below the county spatial scale. Aggregated county data are presented in this publication
